# Functional and microbiological properties of spirulina soybean tempeh flour modified by heat-moisture treatment and annealing

**DOI:** 10.1038/s41598-025-22096-x

**Published:** 2025-10-31

**Authors:** Vira Putri Yarlina, Naydelline Nitema Warae, Herlina Marta, Nur Akmalia Hidayati, Heni Radiani Arifin, Putri Widyanti Harlina, Mohd Nizam Lani

**Affiliations:** 1 Food Technology Program, Food Industrial Technology Department, Faculty of Agro-Industrial Technology, Universitas Padjadjaran, Bandung, 45363 Indonesia; 2 Research Center for Applied Microbiology, BRIN, Bogor, 16911 Indonesia; 3 Department of Fisheries and Food Science Faculty, Universiti Malaysia Terengganu, Terengganu, 21030 Malaysia; 4 Adjuct Professor Faculty of Agro-Industrial Technology, Universitas Padjadjaran, Bandung, 45363 Indonesia

**Keywords:** Spirulina-tempeh flour, Heat-moisture treatment, Annealing, Functional properties, Physicochemical properties, Biochemistry, Biotechnology, Environmental sciences, Microbiology

## Abstract

Spirulina, known for its high protein content, can be developed into tempeh and further processed into flour for bakery products. However, the direct use of spirulina-tempeh flour as a premix presents challenges, particularly in achieving stable volume and texture. This study aimed to determine the most effective modification method for improving the characteristics of spirulina-tempeh flour. Accordingly, two modification techniques were applied: Heat-Moisture Treatment (HMT) and annealing. Following this, statistical analysis was conducted using one-way Analysis of Variance (ANOVA) with Duncan’s Multiple Range Test (DMRT) (*p* < 0.05), and the De Garmo method was used to identify the best treatment. Overall, the results revealed that HMT was the most effective method in enhancing flour properties. In particular, the HMT-modified flour exhibited the following values: moisture content 4.82% db, fat content 27.63% db, Ash Content (AC) 2.35% db, protein content 41.40% db, Water Absorption Capacity (WAC) 1.93 g/g db, Oil Absorption Capacity (OAC) 0.38 g/g db, syneresis 77.49% db, swelling volume 4.85 mL/g db, solubility 0.20% db, antioxidant activity (IC₅₀) 49.998 ppm, starch content 1.903% db, and amylose content 0.007% db. Meanwhile, microbiological properties further indicated a Standard Plate Count (SPC) of 2.74 × 10⁴ CFU/g. In conclusion, HMT effectively improved the functional, physicochemical, and microbial characteristics of spirulina-tempeh flour, making it more suitable for use in bakery products.

## Introduction

Microalgae have been recognized as a food source for centuries, with historical records demonstrating their use as early as 1500 BC^1^. Spirulina (*Arthrospira platensis*) has recently gained global recognition as a traditional food supplement and as a novel nutraceutical and functional ingredient applicable across diverse food systems. Notably, spirulina and other microalgae are relatively easy to cultivate, requiring fewer natural resources than conventional crops. Specifically, spirulina contains up to 60% to 70% high protein with complete essential amino acids, vitamins, minerals, and bioactive pigments such as phycocyanin, chlorophyll, and carotenoids. These characteristics make spirulina a valuable nutritional supplement and position it as a promising ingredient in the development of functional foods^1,2^. Furthermore, spirulina cultivation requires relatively low resources and has a small ecological footprint, making it attractive in the context of sustainable food production and global nutrition security.

Spirulina (*A. platensis*) has been granted Generally Recognized as Safe (GRAS) status by the US Food and Drug Administration, with a rich source of nutrients with notable antioxidant, anti-inflammatory, and immunomodulatory properties^3,4^. At the same time, it contributes to improved metabolic health, oxidative stress protection, and immune function. Such qualities make it a desirable candidate for the creation of functional foods and nutraceuticals intended to address contemporary health issues, in addition to being a promising dietary supplement.

The modern food industry is shifting toward innovations that integrate health-promoting ingredients into convenient, everyday products. Following this, spirulina has become a crucial ingredient in the modern food market, incorporated into various items, such as pasta, bread, snacks, yogurt, and beverages, through biotechnological breakthroughs. One such advancement is the development of premix flours, designed to meet consumer demands for convenient, ready-to-use, and nutrient-rich products^5^. Within this context, the use of plant proteins, micronutrients, and microalgae such as spirulina to fortify flour-based products has become a common strategy to improve nutritional value. In line with this, adding spirulina to flour systems is becoming more common as it makes baked goods and other flour-based foods more nutritious, increases the number of bioactive compounds, and improves their functional potential. Moreover, studies have demonstrated that adding spirulina to breads, drinks, and dairy products can make them more antioxidant-rich. It can also help businesses diversify their products and keep up with health-conscious consumer trends.

Another research suggests that using spirulina as a fortifying ingredient in various food systems has positive effects. Gluten-free breads fortified with spirulina, for instance, illustrated improved antioxidant activity and volatile compound profiles, demonstrating that spirulina enrichment can boost bioactive compound levels and improve prebiotic potential in flour-based products. Additionally, similar advantages were noted in pasta and bakery systems, where the addition of spirulina enhanced functional qualities and increased protein content without sacrificing sensory acceptance. All of these results highlight spirulina’s potential as a flexible fortifier that improves the nutritional content and practicality of foods made with flour^6^. Beyond bakery systems, spirulina fortification has also been explored in beverages and dairy products, further demonstrating its versatility. A study on the physicochemical properties of a spirulina, wheat germ, enriched high-protein functional beverage based on pear-cantaloupe juice revealed significant improvements in nutritional and functional attributes^7^. Similarly, spirulina has been reviewed for its applications in dairy products, where it improves nutritional quality and contributes to sustainability through potential roles in recycling dairy by-products^8^. Together, these findings emphasize spirulina’s broad applicability as a fortification ingredient across diverse food categories.

Tempeh, a traditional Indonesian fermented soybean product, offers an additional promising avenue for the application of spirulina. Fermentation by *Rhizopus* spp. makes proteins and bioactive substances easier to digest and absorb, while also lowering the levels of anti-nutritional agents. Fermentation processes, such as those applied in tempeh production, can further enhance the bioavailability and stability of spirulina’s nutrients^9^. In essence, making flour from tempeh with spirulina in it is suitable for shelf life, variety, and convenience of use in recipes that call for more than one type of flour^10^. However, due to its high protein and nutrient density, using tempeh flour directly in baked goods can lead to technological issues, such as decreased loaf volume, changed crumb structure, and increased microbial susceptibility^11–13^. Correspondingly, these limitations highlight the need for functional modifications to optimize spirulina-tempeh flour as a fortification ingredient without compromising its nutritional values.

Spirulina-tempeh flour presents similar challenges to other legume- and algae-enriched flours, where high protein and bioactive content can interfere with starch gelatinization, water absorption, and dough rheology, resulting in reduced loaf volume and altered texture. These limitations are directly associated with research on modified flours, which indicates that hydrothermal treatments such as Heat-Moisture Treatment (HMT) and annealing enhance the functionality of cereals and legumes. Furthermore, research on corn, rice, and sweet potato flours indicates that HMT reduces excessive swelling and syneresis by promoting stronger molecular connections, and annealing improves hydration and solubility through gradual structural reorganization. In addition, HMT involves applying high temperatures under restricted moisture, enhancing structural stability without destroying starch granules, while annealing promotes molecular rearrangement that reduces swelling and amylose leaching^13,14^. Both methods have been widely studied in cereals and legumes, with reports demonstrating improvements in water and oil absorption, solubility, syneresis, and overall flour performance in food applications. Therefore, it is anticipated that using these methods on spirulina-tempeh flour will balance its richness in nutrients and bioactive compounds with enhanced technological qualities, enhancing its potential as a sustainable and adaptable ingredient for baking applications. Concurrently, the study aimed to compare the effects of HMT and annealing on the physicochemical, functional, microbiological, and structural properties of spirulina-fortified tempeh flour, with the best treatment utilizing the De Garmo effectiveness index.

.

## Methods

### Materials

The materials employed in this study include soybeans, tempeh, and spirulina-fortified soybean tempeh, all of which were sourced from Rumah Tempe Indonesia (RTI) (Bogor, West Java, Indonesia). Acetic acid (CH₃COOH), cooking oil (for OAC), DPPH, distilled water (aquades), ether, boric acid, hexane, hydrochloric acid (25%), Kjeldahl tablets, methanol, mixed indicator of methyl red and bromocresol green, NaCl 0.85% dilution solution, NaOH, Neutral Red, Plate Count Agar (PCA) medium (Oxoid Ltd., Hampshire, UK), concentrated sulfuric acid (H₂SO₄), and 95% ethanol.

## Spirulina tempeh production

Tempeh was prepared following Romulo and Surya (2021)^15^ with a modification. The protocol included sorting, soaking, dehulling, boiling, and fermentation. At the same time, the slight modification involved adding 1% (w/w) *Spirulina* (*A. platensis*) powder to the cooled soybeans before inoculation.

The soybeans were initially sorted and rinsed using clean water, then boiled at 100 °C for 30 min. Subsequently, in the preliminary boiling step, the beans were soaked at room temperature for approximately 20 to 22 h. Once soaking was complete, the hulls were manually removed, followed by a second boiling phase lasting 40 min. Once dehulled, the soybeans were drained and brought to ambient temperature (25 °C to 27 °C) before further processing. *Spirulina* (*A. platensis*) powder was incorporated into the cooled soybeans [1% (w/w)], along with a commercial tempeh starter culture containing *Rhizopus oligosporus*. The mixture was subsequently packed into perforated Polyethylene (PE) bags to facilitate aeration. The fermentation process is conducted within 30 °C to 37 °C with 70% to 85% relative humidity for 46 to 48 h^15^ until the surface of the product is uniformly covered with white fungal mycelia.

## Production of spirulina-enriched tempeh flour

The method followed Mursyid et al. (2014)^16^. Accordingly, the fermented tempeh was sliced into thin pieces and placed on trays, followed by drying in an oven (Shel Lab SMO28-2) at 50 °C for 24 h. Then, the tempeh was milled and sieved using an 80-mesh sieve for consistent particle dimensions of flour.

## HMT modification of spirulina-enriched tempeh flour

HMT spirulina tempeh flour by Marta et al. (2022)^17^ with modification. The native spirulina-tempeh flour was adjusted to 30% moisture content. Distilled water (A) was calculated using the formula:

$$\:A={W}_{2}-{W}_{1},$$

1 $$\:A=\left\{\frac{\left(100\:\%-\:{KA}_{t}\right)\:x\:Wt}{100\:\%-\:{KA}_{HMT}}\right\}-{W}_{1}$$.

Notes:

A = volume of distilled water to be added (mL).

KAₜ = initial moisture content of flour (%).

Wₜ = initial weight of flour (g).

KA_HMT_ = desired moisture content for HMT (%).

W1 = initial weight of flour before treatment (g).

The flour was subsequently placed in an airtight container and stored in a refrigerator (4 °C) for 21 h to equilibrate the water content. The modified flours were subjected to heat treatment in an oven (Memmert UNB-300) at 85 °C for 18 h. The treated flour was subsequently dried in an oven (Shel Lab SMO28-2) at 50 °C for 24 h, milled, and sieved through ≥ 80-mesh sieve. Concurrently, the modified flour was vacuum-packed in moisture-resistant plastic bags.

## Annealing modification of Spirulina-Enriched Tempeh flour

The annealing process of modified spirulina-tempeh flour by Z. Zhang (2023)^13^, with certain procedural modifications applied. In particular, the native spirulina tempeh flour was adjusted to 55% of moisture content by adding distilled water. The hydrated flour was placed into a Schott bottle and incubated in a water bath at 50 °C for 24 h to allow molecular rearrangement. After incubation, the modified flours were dried in a drying oven (Shel Lab SMO28-2) at 50 °C for 24 h. The dried modified flour was subsequently ground, sieved, and stored in a vacuum-sealed package.

### Proximate, Starch, and amylose content

The proximate analysis to determine moisture, protein, fat, fiber, and Ash Contents (ACs) was conducted following the Official Methods of Analysis (AOAC) (2005)^18^ standard procedures and supported by more recent applications in soy and tempeh-based products^19,20^. Meanwhile, moisture content was determined using the AOAC 934.01, protein content (AOAC 978.04), fat content (AOAC 930.09), and AC (AOAC 930.05). Correspondingly, the starch content was analyzed according to Hernandez-Carmona et al. (2017)^21^, and the amylose content was measured by Sujka and Jamroz (2013)^22^.

## Antioxidant activity (DPPH Method)

The antioxidant activity was evaluated following the method of Dhillon et al. (2020)^23^ with slight modifications. A 100 mg was extracted with 10 mL of 80% methanol and stirred at 200 rpm for 2 h (IKA Vortex Genius, Germany). The mixture was then centrifuged (Hermle, Germany) at 6,000 rpm for 10 min at 25 °C (repeated twice). The supernatants were pooled and filtered through a 0.45 μm Acrodisc membrane filter (Pall Corporation, USA). The filtrate was either used immediately for analysis or stored at − 20 °C. DPPH assay with 100 µL of the extract or standard solution was mixed with 3.9 mL of DPPH reagent (6 × 10⁻⁹ mol/L), and the absorbance was recorded at 515 nm after 0 and 30 min of incubation using a Ultraviolet-Visible (UV-Vis) spectrophotometer (Shimadzu UV-1800, Japan).

2 $$\:\text{\%}\:Antioxidants=\:\left[1-\:\left[\frac{Absorbance\:value\:when\:t=30}{Absorbance\:control\:value}\right]\right]\:\times\:\:100$$.

## Water and oil absorption capacity

Note that 1 g of spirulina-tempeh flour was placed into a centrifuge tube and mixed with 10 mL of distilled water by Marta et al. (2024)^24^ with slight modifications. Oil Absorption Capacity (OAC) was combined with 10 mL of cooking oil. Each mixture was vortexed for 30 s (Vortex 3, Germany) and allowed to stand at room temperature for 1 h. The tubes were centrifuged (Hermle Z 206) at 3,500 rpm for 30 min. As such, Water Absorption Capacity (WAC) was counted as the volume of water absorbed per gram of dry sample, while OAC referred to the oil absorbed per gram of dry sample.

### Freeze-thaw Stability/Syneresis

An 8% (w/v) flour/starch suspension was prepared and heated in a water bath at 95 °C for 30 min to form a paste. Consequently, the paste was cooled to 50 °C, and 20 g was transferred and placed into pre-weighed 50 mL centrifuge tubes. These tubes were sealed and stored at 4 °C for 24 h, followed by freezing at − 20 °C for 48 h. The samples were thawed at room temperature for 4 h and centrifuged at 3,500 × g for 15 min using Hermle Z 206 A (Germany). The supernatant was separated and weighed, and the amount of liquid as syneresis, reflecting the freeze-thaw stability of the flour paste. This method is described by Marta et al. (2024)^24^, with slight adjustments. The freeze-thaw stability was calculated using the formula:

3 $$\:Syneresis\:\left(\%\right)=\left(\frac{Supernatant\:\left(g\right)}{Total\:weight\:of\:suspension\:in\:the\:tube\:\left(g\right)}\right)\times\:100$$.

#### Swelling volume and solubility

Correspondingly, 0.35 g of sample flour was placed into a centrifuge tube and mixed with 12.5 mL of distilled water, following Marta et al. (2024)^24^. The suspension was vortexed until homogeneous and heated in a water bath at 90 °C for 20 min. The tubes were rapidly cooled in cold water and centrifuged (Hermle Z 206 A) at 3,500 rpm for 30 min. The sediment volume was determined by recording the volume of sediment after centrifugation. The solubility was measured by collecting the supernatant, which was then transferred to a pre-weighed evaporating dish. The sample was dried at 110 °C for 24 h. Both swelling volume and solubility were calculated using the formulas:

4 $$\:SP\:\left(\%\right)=\frac{{w}_{2}}{wdm}\times\:100$$,

5 $$\:Solubility\:\left(\text{\%}\right)=\:\frac{{w}_{1}}{wdm}\times\:100$$.

The dry matter weight (wdm) was obtained from:

6 $$\:wdm=ws\:\left(1-mc\right)$$,

where.

w₁ = weight of the supernatant (g).

w₂ = weight of the formed gel (g).

ws = sample weight (g).

mc = moisture content of the flour in decimal (wet basis).

### Scanning electron microscope (SEM)

Approximately 1 g of the spirulina-tempeh flour was placed onto an aluminum stub and secured with silver adhesive. The samples were subsequently coated with a thin conductive layer of gold/palladium using a sputter coater at 8 to 10 mA for 10 to 15 min under low-pressure conditions (< 10 torr)^17^. Meanwhile, the microstructure of the flours was scanned using a Scanning Electron Microscope (SEM JEOL JSM IT300, Japan), and images were captured as digital micrographs at a magnification of 2500× and 5000×.

### Starch crystallinity

X-ray diffraction of the flour samples was carried out by a diffractometer (Bruker D8 Advance, Germany). The instrument was operated with a copper (Cu) target at 40 kV and 25 mA, producing Cu Kα radiation at a wavelength of 1.54060 Å. Note that diffraction patterns were recorded over a 2θ range of 10° to 90° at a scan speed rate of 0.2 s per step^17^.

### Fourier transform infrared spectroscopy (FTIR)-ATR

Flour samples were carefully mixed with potassium bromide (KBr) at a ratio of 1:3 (w/w), then pressed and compressed into pellets for analysis. The samples were examined using Attenuated Total Reflectance Fourier Transform Infrared spectroscopy (ATR-FTIR) spectroscopy and a Thermo Scientific Nicolet iS5 spectrophotometer (Massachusetts, USA). Spectra were recorded over the wavenumber range of 4,000 to 400 cm⁻¹, with a resolution of 8 cm⁻¹, and 32 scans per sample. Characteristic absorption peaks were observed at 3,360 to 3,200 cm⁻¹ (O-H stretching), 2,928 to 2,850 cm⁻¹ (C-H stretching), and 1,640 to 1,630 cm⁻¹ (H–O–H bending vibrations), the presence of hydroxyl groups, aliphatic chains, and bound water in the flour matrix^17^.

### Microbiological properties (Total plate count)

Microbiological quality was determined using the Total Plate Count (TPC) method following AOAC 990.12 and ISO 4833-1:2013 standards with slight modification. Here, 25 g of the flour sample was aseptically homogenized in 225 mL of sterile diluent (Buffer Peptone Water (BPW)) to prepare a 10 − ^1^ dilution. Serial tenfold dilutions (10⁻¹ to 10⁻⁶) were made by transferring 1 mL of each previous dilution into 9 mL of fresh sterile diluent. Accordingly, 1 mL from each dilution was pipetted into duplicate sterile petri dishes, followed by the addition of 15 mL of molten PCA (Oxoid, UK), which was mixed thoroughly. The plates were incubated at 35 ± °C for 48 h. The colonies were enumerated after incubation, and only plates containing 30 to 300 colonies were considered for valid microbial counts^25^. Based on the lowest dilution plated, the method’s detection limit was set at 1 × 10² CFU/g, and its upper quantification limit was set at 1 × 10⁶ CFU/g.

### Statistical analysis

The results are reported as mean values ± Standard Deviation (SD) from three independent experiments. Statistical comparisons were performed using one-way Analysis of Variance (ANOVA), followed by Duncan’s Multiple Range Test (DMRT) at a significance level of *p* < 0.05. The data were assessed for compliance with the assumptions of normality (Shapiro-Wilk test) and homogeneity of variance (Levene’s test) to ensure the validity of the analysis. In addition, these analyses were conducted using IBM SPSS Statistics version 27 (IBM Corp., USA). The microbiological evaluation with the optimal treatment is identified using the De Garmo effectiveness index method, in which treatments were scored and ranked based on predefined criteria relevant to assessment.

## Results and discussion

### Antioxidant activity

The antioxidant activities (Table 1**)** indicate that fermentation influences antioxidant capacity, as observed in Sample B (Tempeh Flour). Soybeans naturally contain isoflavone glycosides, compounds in which isoflavones are conjugated with sugar moieties. During tempeh fermentation, *Rhizopus* species secrete β-glucosidase enzymes that catalyze the hydrolysis of these glycosides, converting them into aglycone isoflavones with higher antioxidant potential^26^. Aglycones such as genistein, daidzein, and glycitein exhibit more potent antioxidant activity than the glycoside forms observed in unfermented soybeans. Despite spirulina’s bioactive pigment phycocyanin^27^, Sample C (spirulina tempeh flour) demonstrated no higher activity than tempeh flour alone (B). This may be attributed to the presence of spirulina pigments such as phycocyanin, chlorophyll, and carotenoids, which exhibit light absorption overlapping with the DPPH assay wavelength at 517 nm (phycocyanin at 620 nm^28^; chlorophyll at 430 nm [blue band] and 665 nm [red band]^29^. The interference of pigment absorption within the same wavelength range as DPPH radicals limits the accuracy of DPPH radical scavenging measurements, as proposed by Yeo and Shahidi (2019)^30^. As presented in Table 1, Samples D (HMT Modified Spirulina Tempeh Flour) and E (Annealing Modified Spirulina Tempeh Flour) exhibited significantly higher antioxidant activity than Samples A (Soybean Flour), B (Tempeh Flour), and C (Spirulina Tempeh Flour), indicating the effectiveness of thermal modification^31^. Notably, heat treatments enhance the release of phenolics, isoflavones, and phycocyanin^32^ and may trigger early Maillard reactions that boost antioxidant capacity^33^. Meanwhile, HMT alters starch granules by increasing surface roughness and porosity, thereby enhancing the extraction of bioactive compounds^34^. In annealing treatment, antioxidant compounds are more resistant to degradation due to improved starch granule stability, which enhances the retention of phenolic compounds within the matrix^35^. Furthermore, annealing under optimal conditions (temperature and time) can preserve over 50% of antioxidant activity by balancing structural stability and bioactive compound retention^36^.

**Table 1:** Antioxidants activity.

Sample	Antioxidants (%DPPH)	IC_50_
A	14.46 ± 0.20^a^	48.809^a^
B	28.91 ± 0.00^c^	49.997^a^
C	20.40 ± 0.00^b^	49.982^a^
D	42.77 ± 0.00^d^	49.998^a^
E	42.97 ± 0.00^e^	50.010^a^

All values are means of triplicate determination ± SD. Means within columns with different superscripts are significantly different (p ˂ 0.05). A (Soybean Flour); B (Tempeh Flour); C (Spirulina Tempeh Flour); D (HMT Modified Spirulina Tempeh Flour); E (Annealing Modified Spirulina Tempeh Flour).

 The antioxidant activity was then evaluated using the IC50 parameter, which refers to the concentration of the compound required to reduce 50% of DPPH radicals^29^. In essence, the smaller the IC50 value obtained, the stronger the antioxidant capacity of the compound^29^. The IC50 value (Table 1) obtained for Samples A, B, C, and D indicates a potent antioxidant activity, whereas Sample E exhibits vigorous antioxidant activity^35^.

### Functional properties

The functional properties (Table 2) demonstrate significant differences in Oil Absorption Capacity (OAC) across samples (*p* < 0.05). The OAC of Sample B was not significantly different from that of A, indicating that fermentation did not directly affect OAC. In Sample C, a difference in OAC was observed compared to Sample B. In particular, the spirulina used in Sample C is known to have a high protein content and hydrophobic groups on the protein side chains, which can form a greater number of protein-oil complexes^37^. Consequently, a higher OAC complex is formed compared to Sample B. OAC of *S. platensis* ranges from 2.35 mL/g to 2.85 mL/g^38^. Conversely, Sample D exhibited a significantly lower OAC than the others. HMT at moisture levels ≥ 30% leads to denser particle structures, reducing available space in the matrix to retain oil^39^. As a result, OAC in Sample D decreased due to limited retention capacity. In contrast, Sample E indicated a significant difference from unmodified samples, as the annealing process enhances starch structural stability without substantially disrupting hydrophobic interactions, increasing OAC levels^40^.

**Table 2:** Functional Properties.

Sample	OAC (g/g db)	WAC (g/g db)	SV (ml/g db)	SOL (%db)	Syneresis (%db)
A	0.53±0.04^bc^	1.64±0.07^a^	4.07±0.32^a^	0.43±0.01^e^	80.24±0.24^e^
B	0.58±0.04^c^	2.53±0.06^d^	5.82±0.25^c^	0.17±0.00^b^	71.43±0.03^b^
C	0.48±0.03^b^	2.49±0.02^cd^	6.68±0.58^d^	0.14±0.01^a^	73.28±0.18^c^
D	0.38±0.03^a^	1.93±0.06^b^	4.86±0.11^b^	0.20±0.01^c^	77.50±0.41^d^
E	0.58 ±0.05^c^	2.42 ±0.05^c^	6.45±0.31^cd^	0.24 ±0.01^d^	70.58 ±0.10^a^

All values are means of triplicate determination ± SD. Means within columns with different superscripts are significantly different (p ˂ 0.05). A (Soybean Flour); B (Tempeh Flour); C (Spirulina Tempeh Flour); D (HMT Modified Spirulina Tempeh Flour); E (Annealing Modified Spirulina Tempeh Flour). OAC (Oil Absorption Capacity); WAC (Water Absorption Capacity); SV (Swelling Volume); SOL (Solubility).

Water Absorption Capacity (WAC) values differed significantly among samples (*p* < 0.05). The lower WAC in A reflects unmodified protein-water interactions, influenced by intrinsic (e.g., hydrophilicity, amino acid composition) and extrinsic (e.g., pH, temperature) factors^41,42^. Specifically, fermentation in B increased WAC via protease and lipase activity, which disrupted the protein matrix and improved water uptake^43^. Sample C did not significantly differ from Sample B. Despite spirulina’s high protein content, its WAC is limited (3.37–4.46 mL/g) and temperature-dependent, with poor solubility at room temperature^38^. Annealing (E) maintained a similar WAC to unmodified samples (C) due to stabilized starch structure, limiting water interaction^40^. However, Sample D (HMT) had the lowest WAC. In essence, HMT promotes double-helix formation and amylose-amylose/amylopectin interactions, producing compact granules with restricted hydration^35^. Thus, denser internal bonding and reduced hydroxyl accessibility inhibit water penetration^44^.

Although swelling volume results revealed that Sample E did not differ significantly from B and C, it was substantially different from A and D (*p* < 0.05). Samples A, B, C, and D all differed significantly from one another. Fermentation (B) increased swelling compared to unfermented soy flour (A) due to a more open matrix^43^. In particular, Sample C demonstrated higher swelling than B, attributed to spirulina’s soluble fiber and protein content, which promote water binding and improve swelling volume^45^. HMT (D) reduced swelling due to starch reorganization, stronger molecular interactions, and crystalline disruption^17^. Additionally, annealing (E) illustrated no significant change from C, likely due to starch stability and limited post-fermentation rearrangement^46^.

Solubility results presented significant differences among samples (*p* < 0.05). The decrease in solubility from soybean flour (A) to tempeh flour (B) is attributed to protein breakdown during fermentation, which produces lower molecular weight proteins that migrate easily, yet reduce overall solubility^47^. Notably, Sample C exhibited further reduction in solubility, consistent with Lucas et al. (2017)^48^, who reported that decreased water solubility index with spirulina addition is due to reduced starch gelatinization and increased matrix density. Conversely, HMT modification (D) increased solubility compared to unmodified C, likely due to reduced molecular order and expanded amorphous regions, facilitating starch dissolution^17,49^. Additionally, HMT promotes amylose-amylopectin rearrangement and hydrogen bond disruption, enhancing solubility depending on starch type, moisture content, and heating duration. Annealed flour (E) also presented increased solubility over C. Annealing enhances starch hydration and gradual granule expansion, promoting amylose release^50^. However, improved crystallinity and stronger molecular bonding can eventually limit amylose leaching, balancing solubility effects^49^.

Syneresis values differed significantly among samples (*p* < 0.05), indicating variations in freeze-thaw stability. High syneresis reflects low gel stability and weak water-holding capacity during cold storage^17^. Sample B demonstrated a reduction compared to A due to fermentation, which alters the metabolite profile and breaks down proteins, affecting gel structure and water retention^51^. Meanwhile, Sample C presented an increase in syneresis compared to Sample B. This is attributed to the addition of spirulina, which contains high levels of soluble fiber and protein that can bind water through hydrogen bonding. However, it forms weak gel structures. Consequently, during the freezing process, the bound water is efficiently released by the protein and expelled from the matrix upon thawing, leading to increased syneresis^45^. HMT-treated samples (D) displayed significantly different syneresis. Higher syneresis in HMT-modified samples compared to unmodified ones is attributed to molecular interactions that reduce the water-holding capacity of the starch gel^17^. HMT also reduced water-binding capacity due to increased amylose content and limited granule swelling, as HMT degrades amylopectin and promotes stronger hydrogen bonding, leading to greater water release during thawing^52^. In addition, annealing (E) altered syneresis through changes in granule structure, possibly increasing amylose retrogradation^53^. While short-chain amylose can delay retrogradation, increased molecular associations may lead to coarse gel textures that reabsorb separated water^54,55^.

### Chemical composition

The chemical composition of the native and modified flours is presented in Table 3. The starch reduction in tempeh flour (B) compared to the unfermented soy flour (A) is attributed to fermentation-induced amylase activity, which hydrolyses glycosidic bonds, particularly in amylopectin branches, converting starch into simple sugars^56^. Essentially, in Sample C, the spirulina addition significantly increased total starch due to its branched α-glucan polysaccharides, structurally similar to amylopectin and detectable as such under specific assays^57^. Conversely, Sample D (HMT) significantly reduced starch and amylopectin contents. HMT under low moisture and high temperature promotes internal restructuring and stronger amylose-amylose or amylose-amylopectin interactions, reducing solubility and extractability. It may also form insoluble starch-protein or starch-lipid complexes and degrade starch into undetectable fragments. This aligns with Qi (2024)^58^, who reported that HMT enhances stable double-helical structures that hinder total starch extraction. Additionally, Sample E (annealing) retained higher starch content due to enhanced crystallinity without molecular degradation^59,60^.

**Table 3:** Chemical Composition.

Sample	Starch (%db)	Amylose (%db)	Protein (%db)	Ash (%db)	Moisture (%db)	Fat (%db)
A	4.510 ± 0.0055^d^	0.013 ± 0.0001^d^	38.24 ± 0.35^a^	4.38 ± 0.04^b^	6.50 ± 0.07^c^	27.08 ± 0.03^a^
B	3.222 ± 0.0187^b^	0.010 ± 0.00004^b^	45.93 ± 0.37^c^	2.34 ± 0.06^a^	5.62 ± 0.04^b^	30.00 ± 0.16^d^
C	4.425 ± 0.0250^c^	0.012 ± 0.0010^c^	46.61 ± 0.10^d^	2.43 ± 0.14^a^	6.93 ± 0.10^d^	28.66 ± 0.10^c^
D	1.903 ± 0.0819^a^	0.007 ± 0.0002^a^	41.40 ± 0.34^b^	2.35 ± 0.17^a^	4.82 ± 0.30^a^	27.62 ± 0.45^b^
E	5.935 ± 0.0185^e^	0.016 ± 0.0002^e^	47.01 ± 0.21^d^	2.41 ± 0.01^a^	10.63 ± 0.13^e^	27.80 ± 0.42^b^

All values are means of triplicate determination ± SD. Means within columns with different superscripts are significantly different (p ˂ 0.05). A (Soybean Flour); B (Tempeh Flour); C (Spirulina Tempeh Flour); D (HMT Modified Spirulina Tempeh Flour); E (Annealing Modified Spirulina Tempeh Flour).

The protein content difference between A and B is attributed to fermentation. Raw soybeans contain 36.6 ± 0.7 g/100 g protein^61^. During fermentation, *Rhizopus* produces proteases that hydrolyze soybean proteins (glycinin and β-conglycinin) into simpler, soluble peptides, increasing their detectability by methods such as Kjeldahl^43^. Sample C, with 1% spirulina addition, demonstrated significantly higher protein than B due to spirulina’s high protein content (50–70%)^62^, which contributed significantly despite the small proportion used. Meanwhile, in Sample D, the decrease in protein is linked to HMT thermal effects. Note that proteins are heat-sensitive and prone to denaturation, leading to reduced measurable protein levels^63^. Thermal processing decreases protein content as temperature, duration, and moisture increase^63^. Furthermore, HMT can induce adhesion and agglomeration, promoting interactions among protein, starch, and lipid chains. These interactions form complexes that hinder the release of non-starch components, thereby lowering detectable protein levels^64^. Additionally, annealing treatment (E) resulted in protein content that did not significantly differ from the unmodified sample (C). Annealing, conducted below the gelatinization temperature (40–55% w/w) under high relative humidity (60–80%), involves temperatures insufficient to cause substantial protein denaturation or structural damage, thus exerting minimal effects compared to HMT^65^. Generally, annealing helps preserve protein structure, preventing extensive aggregation or denaturation, enhancing protein stability and functional availability^66^.

AC in Sample A was significantly higher than in the other samples. Ash reflects the total mineral and inorganic content in food and serves as an indicator of purity^67^. Soybean flour typically contains 4.29% to 5% ash^68^. AC may vary significantly depending on the treatment applied^67^. In fermentation treatments (B and C), reductions in ash may result from mineral leaching and microbial degradation during fermentation^69^. Moreover, thermal modifications such as HMT (D) and annealing (E) can also alter ash levels. Additionally, processing methods involving heat, including HMT and annealing, can reduce mineral content in tempeh-derived products^70^.

Moisture content differed significantly across all samples (*p* < 0.05). Sample B exhibited a reduction in moisture content following the fermentation process, with the decrease in moisture content increasing over time. The reduction of moisture content is attributed to the heat generated during fermentation and exposure to air, which promotes water evaporation^71^. In particular, Sample C demonstrated higher moisture content than Sample B due to the addition of spirulina. The presence of insoluble compounds such as spirulina, glute, proteins, starch, and other macromolecules can bind water through deep binding mechanisms^72^. This result is in line with Ersyah et al. (2022)^73^, who reported increased moisture content in dry noodles enriched with spirulina. HMT (D) significantly reduced the moisture content. HMT, conducted under low moisture (10–35%) and high temperature (80–120 °C), strengthens molecular bonding without causing gelatinization^44^. This leads to denser granule structures, limiting water uptake and resulting in lower moisture levels compared to native starch^35^. In addition, annealing (E) significantly increased moisture content due to the rearrangement of starch granule crystalline structures, creating more open regions that enhance water binding in both amorphous and crystalline zones^74^. As such, water absorption rises with stronger amorphous-crystalline interactions and is influenced by the water-to-temperature ratio during annealing^74^.

The increase in fat content in Sample B compared to Sample A is attributed to fermentation, which enhances lipid availability^75^. During tempeh fermentation, lipase enzymes hydrolyse complex lipids into glycerol and fatty acids, making them more detectable in chemical analyses^75^. The difference in fat content between Samples B and C is attributed to the addition of spirulina^76^. Spirulina contains relatively low-fat levels, which contribute to the overall reduction in fat content in Sample C. It also contains approximately 7% fat, and its incorporation into tempeh products can lower total fat levels^76^. Spirulina’s fat content is considered more functional than quantitative, as it primarily consists of health-beneficial unsaturated fatty acids. Moreover, thermal modifications such as HMT (D) and annealing (E) also influenced fat content. In HMT, adhesion and agglomeration promote the formation of starch-lipid complexes that hinder the release of non-starch components^63^. These complexes are resistant to both extraction and digestion. This results in significantly lower measurable fat content. In Sample E, annealing also led to a decrease in fat content compared to the non-modification sample (C), due to strengthened starch chain interactions, improved crystalline regions, and the formation of amylose-lipid complexes^77^. These complexes are resistant to solvent and enzymatic action, thereby limiting lipid release and digestion, and consequently reducing detectable fat levels^77^.

#### Granule morphology

Soybean flour (A) **(**Table 4**)** granules are irregular, fragmented, and lack the defined spherical or polygonal shapes observed in cereal starches such as corn or rice, which is consistent with previous findings^78^. Notably, the surfaces are rough, porous, and cracked due to high mechanical stress during dry milling. With a high protein content and relatively low starch, the granules are often coated or embedded in amorphous protein-carbohydrate matrices^78^. As a result, starch granules are not prominent, and distinct granular patterns commonly observed in starchy sources are absent. Microbial fermentation alters the microstructure of soybean particles, leading to fragmentation and the formation of irregular aggregates^79^. Additionally, the degradation of native structures during fermentation promotes the formation of amorphous protein-carbohydrate complexes with increased surface area and a smoother overall morphology^80^.

The HMT modified sample suggests that the particles exhibit a more compact and irregular shape, with smoother yet fractured surfaces in some areas. However, intact starch granules are not visible, indicating morphological disruption. These changes are attributed to heat and limited moisture during treatment, which promote granule swelling, partial gelatinization, and surface melting^17^. As a result, starch merges into dense, amorphous protein-carbohydrate matrices, altering the flour’s structure and potentially its functional properties. At the same time, SEM analysis of annealed spirulina soybean tempeh flour presented compact, smooth granule surfaces with intact morphology due to the low annealing temperature (50 °C), which is in line with Puelles-Román et al. (2021)^81^, Y. Zheng et al. (2023)^82^. Additionally, the presence of a distinct, spherical granule may indicate the formation of ordered protein-carbohydrate structures.

**Table 4:**
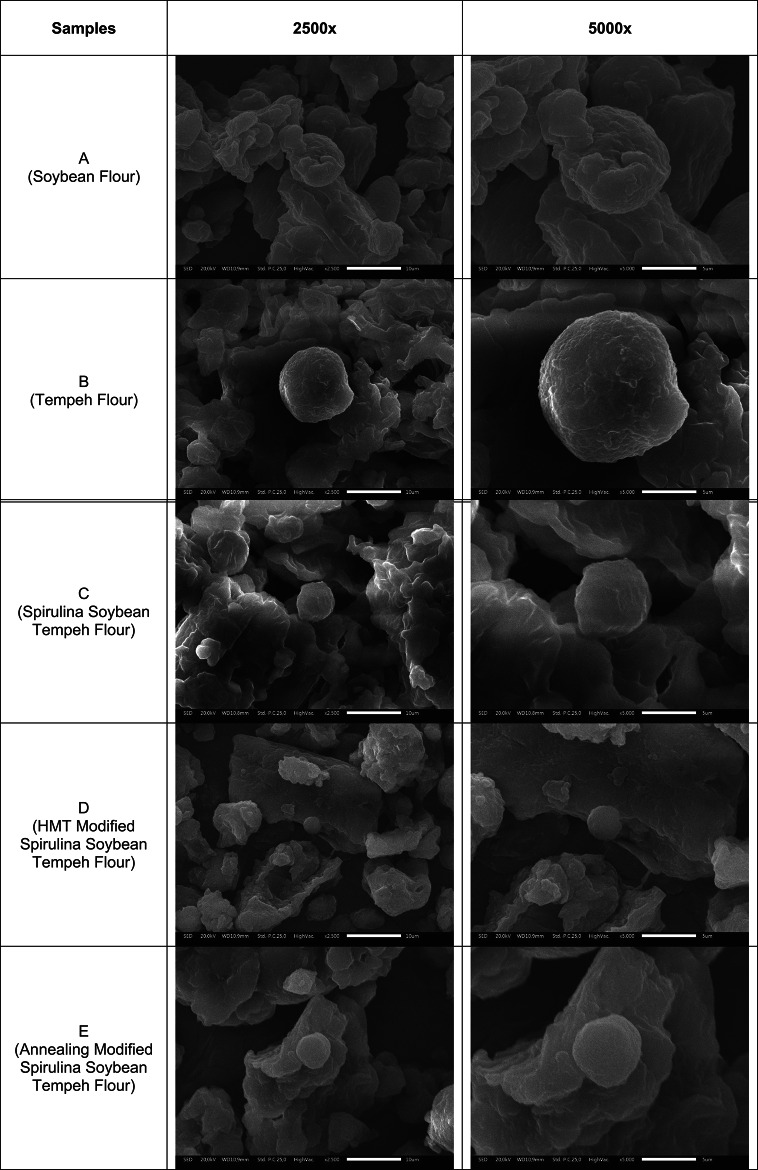
Granule morphology at 2500x magnification and 5000x magnification.

### Starch crystallinity

**Fig. 1:**
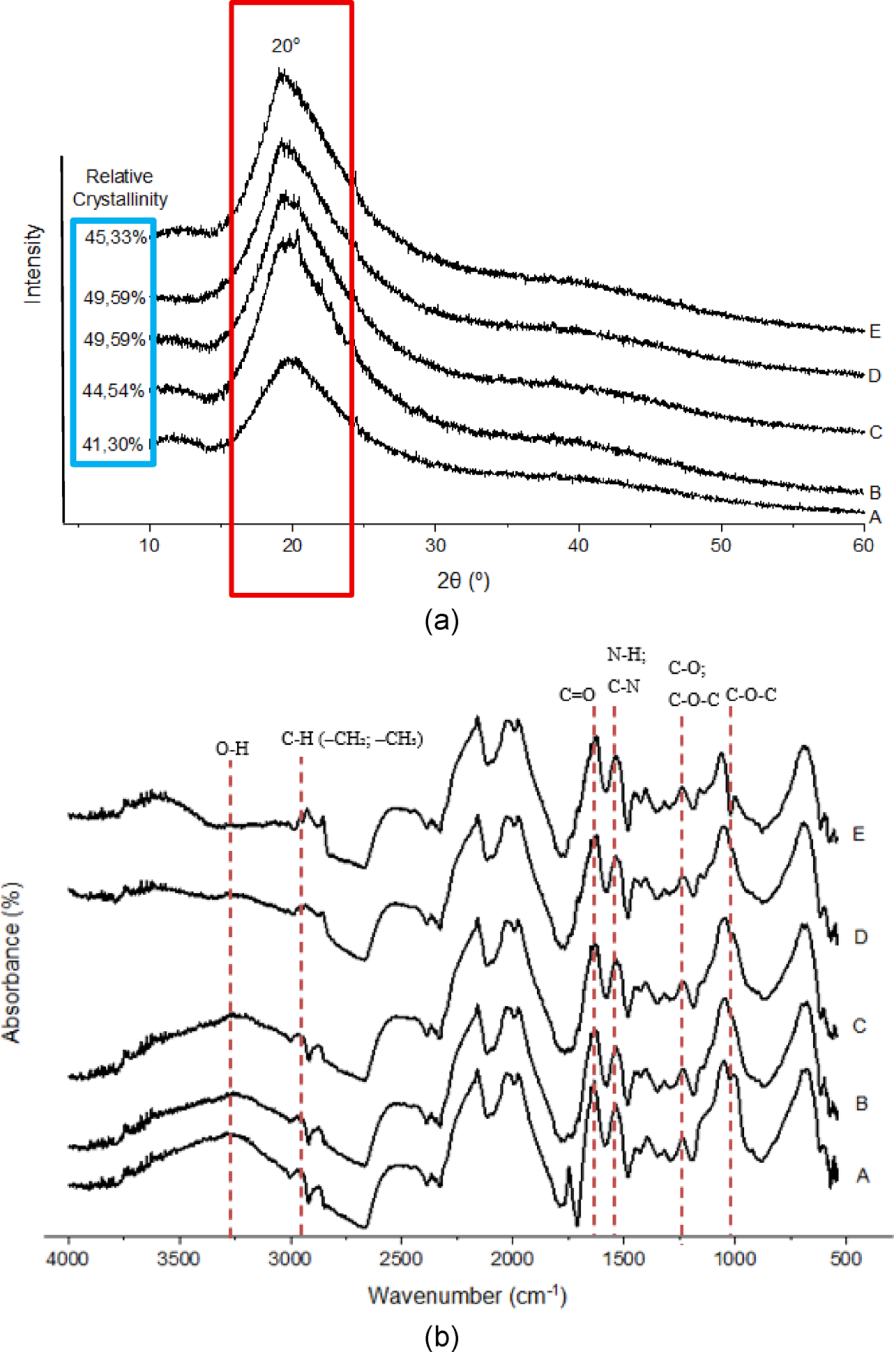
(**a**) Starch crystallinity and relative crystallinity of native and modified flours; (**b**) FTIR spectra of native and modified samples. A (Soybean Flour); B (Tempeh Flour); C (Spirulina Tempeh Flour); D (HMT Modified Spirulina Tempeh Flour); E (Annealing Modified Spirulina Tempeh Flour).

The results, as shown in Fig. 1, indicate that the XRD patterns of the five samples exhibit distinct peaks in the 2θ range of approximately 15° to 25°, with the most intense peak occurring at around 20°. This diffraction pattern is characteristic of type A starch crystallinity, commonly observed in cereal starches, such as those from soy, maize, and wheat. In particular, type A starch typically exhibits diffraction peaks at around 15°, 17°, 18°, and 23°, which may merge into a broader peak around 20° in samples with lower crystallinity or slight noise^83^. The value of soybean flour (A) was the lowest at 41.30%. In tempeh flour (B), the value increased to 44.54%. Adding spirulina (C) increased crystallinity to 49.50%, and heat–moisture treatment (D) gave the highest RC at 49.59%. Annealing (E), on the other hand, reduced the value slightly to 45.53%.

Therefore, all samples are presumed to exhibit type A crystallinity. This result differs from Stevenson et al. (2006)^84^, who suggested that most legume starches exhibit a type-C crystalline structure. These discrepancies indicate that X-ray diffraction patterns are highly dependent on various factors, including the plant variety, agro-ecological conditions, and the pretreatment applied to the samples. Additionally, the absence of a peak at the 2θ angle of 6.5° indicates that the sample does not exhibit B-type crystallinity. This interpretation is supported by Kaptso et al. (2014)^83^, which posited that bambara groundnut starch is a type-A crystalline, and by Liu et al. (2019)^57^, which also identified it as a type-A crystalline on other legumes, such as cowpea, black beans, and carioca. Moreover, the fermented sample (B) exhibits a similar trend to Sample A, as fermentation did not alter its crystalline structure, consistent with Li et al. (2024)^85^. Sample C also demonstrates no difference in crystalline type, as the added spirulina did not disrupt the structure, which is consistent with Jia et al. (2024)^86^. Similarly, HMT-modified and annealing-modified samples (D & E) did not exhibit changes in diffraction patterns. These results imply that the crystal form remains unaffected by the applied modifications. Similarly, these findings align with Lucas et al. (2017)^48^, which confirms that HMT and annealing do not affect “A”-type crystalline pattern of maize starch. The effect of temperature and moisture conditions on starch crystallinity is influenced by the source and the treatment conditions, which indicates that not all conditions can lead to changes in crystallinity^48^.

Fermentation-modified Sample B (tempeh flour) exhibited an increase in relative crystallinity. The increase in relative crystallinity observed is likely due to fermentation-induced selective hydrolysis of amorphous regions within the starch granules^87^. Enzymes produced by *Rhizopus* during fermentation preferentially degrade these less-ordered areas, leaving the crystalline regions intact. As a result, the proportion of crystalline structure increases relative to the total granule. This enhances the relative proportion of intact crystalline domains, as reported in analogous studies on wheat^87^ and rice flour^88^ fermentation. Essentially, incorporating spirulina powder (at a specific concentration) can increase its relative crystallinity, implying that spirulina addition did not disrupt the starch’s crystalline structure and may improve it. Specifically, spirulina contains functional compounds such as proteins, polysaccharides, and antioxidants that can interact with starch granules. These compounds may stabilize or promote molecular alignment in the starch matrix during drying or processing. Rather than disrupting the crystalline regions, spirulina may help reinforce hydrogen bonding, which enhances the crystalline structure. This finding is also in line with J. C. Chung and L. S. Lai (2023)^40^, that suggested that incorporating spirulina can increase the relative crystallinity of rice starch to approximately 48.13%.

The HMT modified sample demonstrates no differences compared to the unmodified Sample C in the number of relative crystallinities, suggesting that the HMT did not substantially alter the crystalline structure of the starch^89^. This result may be attributed to the inherent stability of the native starch’s crystalline regions, which were not significantly reorganized under the applied treatment conditions^89^. In addition, the relative crystallinity number is highly dependent on factors such as moisture content, temperature, and time during the HMT treatment^89^. Annealing modified sample (E) demonstrates a decreased number of relative crystallinities compared to Sample C. The reduction of relative crystallinity indicated that disruption of the crystalline structure and rearrangement of starch molecules coincided with annealing treatment^82^. The other effect of annealing on crystallinity, as reported by Hong et al. (2022)^87^, indicates that crystallinity decreases when the annealing temperature exceeds the optimal ceiling, even if it remains below the gelatinization onset.

#### Functional groups and molecular order degree (FTIR-ATR)

The FTIR **(**Fig. 1**)** spectra of all samples (A-E) exhibited similar peak positions, indicating that no new functional groups were introduced during fermentation, spirulina addition, HMT, or annealing modification^17^. All spectra (A-E) exhibited similar peak patterns, differing only in intensity (absorbance height), yet not in peak positions. This finding, similar to Almeida (2016)^33^, suggests that fermentation significantly influences functional properties without altering the structural architecture of the starch granules, as no new bonds or functional groups were detected. Additionally, HMT and annealing treatments did not form new functional groups, indicating no physical modification^17^.

A broad absorption band around 3,200 to 3,400 cm⁻¹ was observed in all spectra, corresponding to the O–H stretching vibrations of intramolecular or intermolecular hydrogen bonds^90^. The band near 2,930 cm⁻¹ was assigned to C–H stretching vibrations of aliphatic –CH₂ and –CH₃ groups in the polysaccharide backbone^91^. In addition, a weak absorption band was observed around 2,350 cm⁻¹, which is commonly attributed to asymmetric stretching of atmospheric CO₂, and is not considered part of the functional groups present in the sample matrix^91^. Characteristic protein-associated peaks were detected at approximately 1,650 cm⁻¹ (amide I, C = O stretching) and 1,540 cm⁻¹ (amide II, N–H bending and C–N stretching), indicating the presence of protein fractions in the samples, likely originating from the soy-based matrix^91^. In the fingerprint region (1,200–1,000 cm⁻¹), strong bands related to C–O stretching and C–O–C glycosidic linkages were observed, which are typical of starch polysaccharides^91^. Additionally, a small peak at around 1,000 cm⁻¹ corresponded to C–O–C stretching vibrations of α−1→4 glycosidic linkages, confirming the presence of starch molecular structures^92^.

#### Determination of the most suitable treatment

The optimal treatment was selected using the De Garmo effectiveness index method, which allows simultaneous evaluation of multiple parameters by scoring and weighting them according to their functional and nutritional relevance. In this study, parameters such as protein, fat, ash, starch, amylose, amylopectin, WAC, OAC, swelling volume, solubility, and syneresis were considered due to their direct influence on flour performance in bakery applications. Antioxidant activity and moisture content were also prioritized to reflect both health-promoting potential and storage stability.

Each treatment sample (A-E) was assigned Normalized Preference (NP) scores for each parameter, which were then weighted and summed to calculate an overall effectiveness index (Table 5). The higher the index value, the more favorable the treatment. As provided in Table 5, Sample D (HMT-modified spirulina-tempeh flour) achieved the highest total score (0.65), outperforming untreated spirulina-tempeh flour (C, 0.59) and annealing-modified spirulina-tempeh flour (E, 0.60). Furthermore, the superiority of Sample D can be attributed to its balanced profile: high protein (41.40%), favorable WAC (1.93 g/g), acceptable swelling capacity (4.85 mL/g), and strong antioxidant activity (IC50 49.998 ppm), combined with low moisture content (4.82%), which enhances shelf stability.

Based on these considerations, Sample D was identified as the optimal treatment and was subsequently subjected to microbiological evaluation (TPC, SPC) to confirm its safety and suitability for food applications.

**Table 5:** Determination of the best Treatment.

Parameter	A	B	C	D	E
	NP	NP	NP	NP	NP
Protein content	0.00	0.07	0.11	0.04	0.11
Antioxidant	0.11	0.11	0.11	0.11	0.11
Moisture content	0.05	0.02	0.02	0.10	0.00
Starch content	0.00	0.09	0.06	0.03	0.06
Amylose	0.09	0.04	0.04	0.09	0.00
Amylopectin	0.02	0.04	0.04	0.08	0.00
Fat content	0.08	0.03	0.04	0.08	0.00
Ash content	0.00	0.03	0.06	0.02	0.06
Carbohydrate content	0.00	0.02	0.00	0.03	0.05
WAC	0.00	0.05	0.02	0.04	0.04
OAC	0.04	0.01	0.01	0.00	0.05
Syneresis	0.04	0.03	0.03	0.00	0.05
Swelling volume	0.04	0.00	0.04	0.04	0.04
Solubility	0.00	0.02	0.01	0.01	0.03
**Total**	0.46	0.56	0.59	**0.65**	0.60

A (Soybean Flour); B (Tempeh Flour); C (Spirulina Tempeh Flour); D (HMT Modified Spirulina Tempeh Flour); E (Annealing Modified Spirulina Tempeh Flour).

#### Microbiological characteristics based on total plate count (TPC)

The Standard microbial count (SPC) of the spirulina tempeh flour native (C) was 2.90 × 10⁴ CFU/mL, while the HMT-modified flour (D) presented a slightly lower value of 2.74 × 10⁴ CFU/mL. According to Standard National Indonesia (SNI) 3751:2018 for wheat flour, the limit is ≤ 10⁵ CFU/g. The spirulina-tempeh flour (C) and the HMT-modified flour (D) were well within safe limits. Note that the addition of spirulina to food products may help inhibit microbial growth. In line with this, V. Saharan and S. Jood (2020)^93^ reported that increasing spirulina concentrations in bread formulations resulted in lower TPC values compared to the control. This suggests that spirulina contributes to improved microbiological stability in the final product.

Microbial growth in spirulina tempeh flour may be influenced by suboptimal drying temperatures, which are limited to preserve its nutritional content. Essentially, bacteria grow optimally at 50 °C to 60 °C, and the drying process may insufficiently reduce microbial load Hernawati et al., 2018)^94^. In contrast, HMT treatment at 85 °C helped reduce microorganisms, particularly molds.

Unmodified spirulina tempeh flour already contained microbes, consistent with Yang et al. (2022)^95^, who reported that flour products may contain native microbes from storage and processing. Further treatment, such as sterilization, may be necessary to reduce microbial levels in HMT-modified samples.

### Pearson correlation

**Fig. 2:**
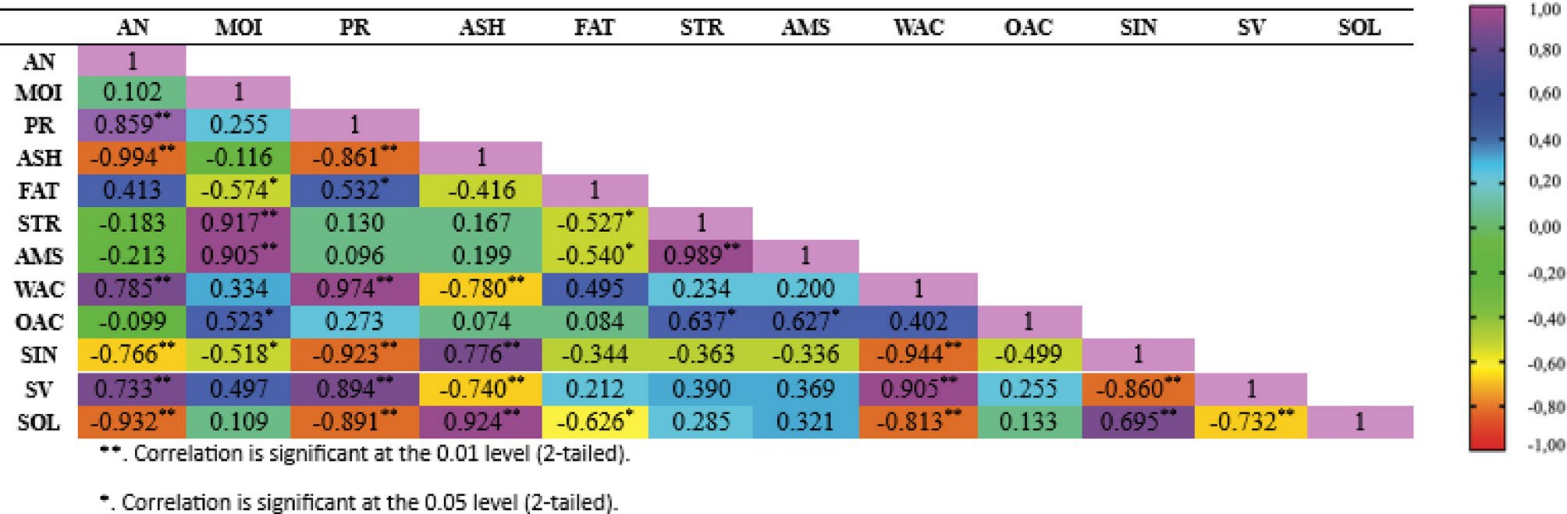
Pearson correlation. A (Soybean Flour); B (Tempeh Flour); C (Spirulina Tempeh Flour); D (HMT Modified Spirulina Tempeh Flour); E (Annealing Modified Spirulina Tempeh Flour); AN (antioxidant activity); MOI (moisture content); PR (protein content); ASH (ash content); FAT (fat content); STR (starch content); AMS (amylose); WAC (water absorption capacity); OAC (Oil Absorption Capacity); SIN (freeze-thaw syneresis); SV (swelling volume); SOL (solubility).

As observed in Fig. 2, the study revealed that protein content was significantly correlated with several physicochemical and functional parameters. Pearson Correlation demonstrated strong positive correlations with WAC (*r* = 0.974, *p* < 0.01) and swelling volume (SV) (*r* = 0.894, *p* < 0.01), and strong negative correlations with ASH (*r* = − 0.861, *p* < 0.01), syneresis (SYN) (*r* = − 0.923, *p* < 0.01), and solubility (SO) (*r* = − 0.891, *p* < 0.01). WAC is influenced by protein denaturation, as denatured proteins have lower solubility yet greater water-binding capacity^96^. Proteins also bind water through polar groups such as carbonyl, hydroxyl, amino-carboxyl, and sulfhydryl groups^97^. The positive correlation between PC and SV is attributed to the ability of hydrated proteins to occupy more space, thereby increasing swelling volume^98^. Higher protein content also enhances water retention, reducing syneresis due to the hydrophilic nature of proteins^99^. Moreover, “protein solubility is influenced by the balance of polar and nonpolar groups and environmental factors such as pH, temperature, and processing conditions^96^.

Moisture content indicated a strong positive correlation with starch (*r* = 0.917; *p* < 0.01) and amylose content (*r* = 0.905; *p* < 0.01). This suggests that higher moisture facilitates starch granule swelling and solubilization, enhancing the release and extractability of starch and amylose from the matrix, thus increasing their measured levels. This is supported by Yang et al. (2021)^100^, who noted that optimal moisture levels promote amylose release and extraction, while also modifying starch structure and influencing its overall physicochemical properties.

Antioxidant activity displayed strong positive correlations with WAC (*r* = 0.785; *p* < 0.01) and swelling volume (*r* = 0.733; *p* < 0.01), indicating that antioxidant compounds such as phenolics and bioactive peptides enhance the matrix’s ability to absorb and retain water through hydrogen bonding^101^. Huo et al. (2025) reported that polyphenols form hydrogen bonds with starch and water in food systems, and that low-molecular-weight peptides with polar groups exhibit higher water-holding capacity. Conversely, antioxidant activity presented strong negative correlations with ASH (*r* = − 0.994; *p* < 0.01), syneresis (*r* = − 0.766; *p* < 0.01), and solubility (*r* = − 0.932; *p* < 0.01). Increased antioxidant activity was associated with reduced free mineral content and improved gel stability (indicated by lower syneresis). This includes a denser, less soluble matrix structure, minimizing the release of soluble compounds during storage or heating.

ASH exhibited strong positive correlations with syneresis (*r* = 0.776; *p* < 0.01) and solubility (*r* = 0.924; *p* < 0.01), indicating that higher mineral levels promote water release and component solubilization, likely by destabilizing starch-protein gels. As reported by Gong et al. (2024)^102^, Na⁺ ions weaken gel structure, inhibit retrogradation, and increase syneresis and solubility. Conversely, ASH correlated negatively with swelling volume (*r* = − 0.740; *p* < 0.01) and WAC (*r* = − 0.780; *p* < 0.01), suggesting that excess minerals reduce water absorption and expansion. According to G. Xiang et al. (2023)^64^, mineral ions may bind to hydroxyl groups, disrupt hydrogen bonding, and produce a more rigid matrix that limits swelling.

WAC illustrated a strong positive correlation with swelling volume (*r* = 0.905; *p* < 0.01), indicating that higher water absorption enhances granule expansion. According to Somala Tatiana et al. (2021)^103^, water uptake promotes hydrogen bonding with starch polar groups, leading to swelling. WAC also correlated negatively with syneresis (*r* = − 0.944; *p* < 0.01) and solubility (*r* = − 0.813; *p* < 0.01), suggesting that higher WAC contributes to a more stable gel matrix with reduced water release and solubility. Hoover et al. (2003)^52^ reported similar findings, where increased WAC reduced syneresis in esterified chickpea starch.

Syneresis correlated positively with solubility (*r* = 0.695; *p* < 0.01), indicating that loosely structured gels tend to release more water. Nagasaka & Taneya (2000)^104^ posited that lower syneresis is associated with higher gel modulus, reflecting better water retention. A strong negative correlation was also observed between syneresis and swelling volume (*r* = − 0.890; *p* < 0.01), suggesting that greater swelling capacity supports water retention^105^. Singh et al. (2006) reported that a significant negative correlation exists between syneresis and swelling after 72 h.

Swelling volume revealed a strong negative correlation with solubility (*r* = − 0.732; *p* < 0.01), implying that greater expansion reduces solubility. This may be due to partial leaching of starch molecules from granules after peak swelling, as reported by Chisenga et al. (2019)^106^.

## Conclusion

Based on the results, HMT modification was the most effective treatment in enhancing the physicochemical and functional properties of spirulina-tempeh flour. In particular, Sample D (HMT-modified spirulina-tempeh flour) demonstrated improved nutritional quality (protein 41.40%, fat 27.63%, ash 2.35%) and functional attributes (WAC 1.93 g/g, OAC 0.38 g/g, swelling volume 4.85 mL/g, and antioxidant activity IC50 49.998 ppm), alongside acceptable microbiological safety (SPC 2.74 × 10⁴ CFU/mL). These properties suggest strong potential for its use as a nutrient-dense flour fortificant in bakery applications, where incorporation at low to moderate levels could enhance the protein and antioxidant profile of bread, pastries, or functional flour mixes without severely compromising dough functionality.

However, several challenges remain. Specifically, sensory quality (flavor, color, texture, and consumer acceptance) was not evaluated, and the long-term shelf-life stability of bakery products formulated with spirulina-tempeh flour also requires further investigation. Hence, addressing these aspects will be critical to determine the maximum feasible incorporation levels and ensure product acceptability in commercial applications.

Future studies should therefore explore optimization of fortification ratios in different bakery matrices, assess consumer perception, and evaluate shelf-life performance. With such advancements, HMT-modified spirulina-tempeh flour holds promise as a sustainable and functional ingredient to support the development of next-generation bakery products with improved nutritional and bioactive properties.

## Data Availability

New data generation or analysis is not reported in this manuscript. The authors must provide detailed information about the datasets used and/or analyzed in this study. Data may be acquired from the corresponding author upon a reasonable request.

## Code availability

No custom code or software application was developed for this research.
